# Hypoxia-Induced LncRNA-MIR210HG Promotes Cancer Progression By Inhibiting HIF-1α Degradation in Ovarian Cancer

**DOI:** 10.3389/fonc.2021.701488

**Published:** 2021-11-25

**Authors:** Ping Liu, Huiqiong Huang, Xiaorong Qi, Ce Bian, Meng Cheng, Lili Liu, Luqi Xue, Xia Zhao, Tao Yi, Yi Quan

**Affiliations:** Department of Gynecology and Obstetrics, Development and Related Diseases of Women and Children Key Laboratory of Sichuan Province, Key Laboratory of Birth Defects and Related Diseases of Women and Children, Ministry of Education, West China Second Hospital, Sichuan University, Chengdu, China

**Keywords:** ovarian cancer, MIR210HG, HIF-1α, hypoxia, progression

## Abstract

LncRNA-MIR210HG plays crucial roles in the progression of diverse cancers. However, the expression and function of MIR210HG in ovarian cancer remains unclear. In the present study, we aimed to determine the expression and function of lncRNA-MIR210HG in ovarian cancer under hypoxic conditions. MIR210HG expression in ovarian cancer cells under hypoxic conditions was determined by qPCR analysis, and the distribution was determined by FISH and qPCR analysis based on cell nucleus and cytosol RNA extraction. Epithelial-Mesenchymal Transition (EMT) assay and human umbilical vein endothelial cell-based tube formation and migration assays were employed to determine the potential function of MIR210HG *in vitro*, followed by establishment of a subcutaneous tumor model in mice. The direct target of MIR210HG was determined by RNA pull-down and western blotting. Furthermore, the expression and clinical correlation of MIR210HG was determined based on malignant tissues from ovarian cancer patients. Our results indicated that MIR210HG was induced by hypoxia, which is HIF-1α dependent and mainly located in the cytosol of ovarian cancer cells. Knockdown of MIR210HG significantly inhibited EMT and tumor angiogenesis *in vitro* and impaired tumor growth in mice. Molecular investigations indicated that MIR210HG directly targets HIF-1α protein and inhibits VHL-dependent HIF-1α protein degradation in ovarian cancer. Further results demonstrated that MIR210HG was upregulated in ovarian cancer tissues and correlated with tumor progression and poor prognosis of ovarian cancer patients. Our study suggests that hypoxia-induced MIR210HG promotes cancer progression by inhibiting HIF-1α degradation in ovarian cancer, which could be a therapeutic target for ovarian cancer.

## Introduction

Ovarian cancer is one of the most challenging diseases in gynecologic oncology, which causes approximately 240,000 new cases and 150,000 deaths every year (1). The most common histological type of ovarian cancer is epithelial ovarian cancer (EOC) (2). Patients with EOC have a 45.6% 5‐year survival rate (3–5), and despite optimal therapy (surgery and platinum-based chemotherapy), the 5-year survival rate in Stages III and IV is between 18–47% (6, 7). Thus, an effort to understand the underlying mechanism of cancer progression is necessary and beneficial for ovarian cancer therapy.

Cancer growth is also associated with the development of limited oxygenation (ranging from nearly anoxia to 8% O2 in the most oxygenated areas *in vivo*), which is often exemplified by an increased amount of proteins from the hypoxia-inducible transcription factor-α family (8, 9). Once a hypoxic and nutrient-poor environment is established, metabolic byproducts and immunosuppressive modulators accumulate (10, 11). Hypoxia is rapidly induced by the O2/prolyl hydroxylases (PHD)/Von Hippel Lindau (VHL) axis, which induces the stabilization of hypoxia-inducible factors and the subsequent activation of a series of genes (12, 13). Accumulating evidence indicates that a hypoxic environment causes metastasis, which is attributed to 90% of cancer-related death (14). Hypoxia is also tightly associated with a poor prognosis and high mortality in patients with ovarian cancer (15). Thus, targeting hypoxia is an effective strategy for cancer therapy.

Long non-coding RNAs (lncRNAs) are a class of non-protein coding RNAs with a length of more than 200 nucleotides. Accumulating evidence has shown that lncRNAs play a crucial role in tumor initiation and progression of diverse cancers (16, 17). MIR210HG is a newly recognized lncRNA that participates in many fundamental biological characteristics of various cancers. Previous studies indicated that MIR210HG promotes cell proliferation and invasion in cervical cancer (18), breast cancer (19), lung cancer (20, 21), and osteosarcoma (22). Upregulation of MIR210HG in malignant tissues correlates with poor prognosis of patients with colorectal cancer (23–25) and hepatocellular carcinoma (26). However, the expression and function of MIR210HG in ovarian cancer remains unclear.

Here, we aimed to determine the expression and function of lncRNA-MIR210HG in ovarian cancer under hypoxic conditions. *In vitro* and *in vivo* function experiments were employed to investigate the potential role of MIR210HG in ovarian cancer. The direct target and underlying mechanism of MIR210HG promoting the progression of ovarian cancer was determined by RNA pull-down, western blotting, and ELISA assays. Furthermore, the expression and potential clinical correlation of MIR210HG in ovarian cancer patients was also investigated. The present study provides solid evidence for understanding the expression and function of MIR210HG in ovarian cancer, which may be a potential therapeutic target for ovarian cancer.

## Methods

### Cell Culture and Treatment

Ovarian cancer cell lines, A2780 and SKOV3, were purchased from American Type Culture Collection (ATCC, USA) and cultured in DMEM (Gibco, MA, USA) containing 10% FBS (Zeta) at 37°C in a humidified atmosphere with 5% CO_2_. For hypoxia condition, A2780 and SKOV3 cells were maintained at 37°C in a humidified atmosphere with 5% CO_2_ and 1% O_2_ (Thermo Fisher, USA). Lentivirus-based shRNA targeting MIR210HG were purchased from GenePharma (Shanghai, China) and used to infect cells with multiplicity of infection (MOI) 20. Puromycin (Beyotime, Beijing, China) was used to select the stably infected cells. siRNA targeting HIF-1α and VHL were purchased from Ribobio (Guangzhou, China) and used to transfect ovarian cancer cells following the instruction of riboFECT CP Transfection Kit (Ribobio, Guangzhou, China). Dimethyloxalylglycine (DMOG) was purchased from Selleck (Catalog number: S7483, Shanghai, China) and used to treat ovarian cancer cells with a final concertation of 4 μM for 24 hours.

### RNA Extraction and Quantitative PCR

Total RNA was extracted from whole ovarian tissues using by TRIzolT (Invitrogen, CA, USA), following as per the manufacturer’s instructions. RNA purity and concentration were monitored using with a NanoDrop ND-2000 spectrophotometer (Thermo Fisher Scientific, CA, USA). One microgram of total RNA was used as template to synthesize cDNA using with the by SuperScript III Reverse transcriptase ion assay (Catalog number: 10880093, Invitrogen, CA, USA). TaqMan Gene Expression Master Mix (Catalog number: 4331182, Thermo Fisher Scientific, CA, USA) was used for real-time Real-time qPCR with a StepOne Real-Time PCR System (Applied Biosystems, CA, USA). GAPDH was used as the loading control. The results of qPCR were analyzed using the conventional ΔΔCt method.

### Western Blotting

Cells were collected and lysed with RIPA buffer (Beyotime, Beijing, China) containing 1% protease inhibitor cocktail on ice and then centrifuged at 12, 000 rpm for 15 min at 4°C. Total 10 μg of total protein was loaded for SDS-PAGE separation and later transferred onto PVDF membranes (Merck Millipore, MA,USA). Then the PVDF membranes were blocked in 5% non-fat milk in TBST buffer at 22-25°C for 1 hour. Primary antibodies against HIF-1α (Catalog number: 36169, CST, MA, USA, 1:800), VHL (Catalog number: 24756-1-AP, Proteintech, Wuhan, China, 1:1000), E-cadherin (Catalog number: 14472, CST, MA, USA, 1:1500), N-cadherin (Catalog number: 13116, CST, MA, USA, 1:1500), Slug (Catalog number: 9585T, CST, MA, USA, 1:1200), Slug (Catalog number: 3879T, CST, MA, USA, 1:1200), and GAPDH (Catalog number: 10494-1-AP, Proteintech, Wuhan, China, 1:5000) were added for incubation at 4°C for overnight. After washing with TBST buffer for 3 times, the HRP-conjugated secondary antibody was added for incubation at 37°C for 1 hour. The antibody-labeled membranes were scanned for signals with iBright (Thermo Fisher, MA, USA) by adding enhanced chemiluminescent (Merck Millipore, MA, USA).

### RNA FISH

A2780 and SKOV3 cells were plated on glass microscope slides at ∼50% confluence and incubated for 24 h in DMEM (Gibco, MA, USA) containing 10% FBS (Zeta) at 37°C in a humidified atmosphere with 5% CO_2_ and 1% O_2_. RNA FISH was performed with the RiboTM Fluorescent *In Situ* Hybridization Kit (Ribobio, Guangzhou, China) following the instruction of manufacturer. The specific probes against U6, 18S and MIR210HG were purchased from Ribobio (Guangzhou, China). Coverslips were mounted with Prolong Gold antifade plus DAPI (Invitrogen) and slides were viewed with BX51 (Olympus, Tokyo, Japan).

### Migration Assay

A2780 and SKOV3 cells (2×10^4^ cells in 100 μl DMEM medium) were seeded into a millicell (8 μm) containing diluted Matrigel (1:5 dilution with DMEM medium). Then the cells were maintained at 37°C in a humidified atmosphere with 5% CO_2_ and 1% O_2_ (Thermo Fisher, USA). Twenty-four hours later, the millicell were collected for crystal violet (Beyotime, Beijing, China) staining. The migrated cells were photographed with BX51 (Olympus, Tokyo, Japan). The number of cells in per frame (4 frames for each group) was analyzed.

### HUVEC-Based Tube Formation Assay and Migration Assay

Cell culture supernatant from A2780 and SKOV3 cells under hypoxia condition were collected and stored at -80°C. HUVECs were purchased from American Type Culture Collection (ATCC, USA) and cultured in EGM-2 medium (Merck Millipore, MA, USA) at 37°C in a humidified atmosphere with 5% CO_2_. HUVEC-based tube formation assay and migration assay were performed as previous study indicated (27). For HUVEC-based tube formation assay, 50 μl Matrigel was added into each well of 96-well plate. Then 2×10^4^ HUVECs in 100 μl conditional culture medium from A2780 and SKOV3 cells were added, and 4-6 hours later, the tubes in each well were photographed with an inverted microscope (Nikon, Tokyo, Japan). The number of branch points in each well were analyzed. For HUVEC-based migration assay, 2×10^4^ HUVECs in 100 μl conditional culture medium from A2780 and SKOV3 cells were seeded into a millicell (8 μm) containing diluted Matrigel (1:5 dilution with DMEM medium). Then the cells were maintained at 37°C in a humidified atmosphere with 5% CO_2_ and 1% O_2_ (Thermo Fisher, USA). Twenty-four hours later, the millicell were collected for crystal violet (Beyotime, Beijing, China) staining. The migrated cells were photographed with BX51 (Olympus, Tokyo, Japan). The number of cells in per frame was analyzed and four frames were included in each analysis.

### Enzyme-Linked Immunosorbent Assay

Cell culture supernatant from A2780 and SKOV3 cells under hypoxia condition were collected and used for ELISA detection following the instructions of VEGF-ELISA kit (NeoBioscience, Shenzhen, China).

### Chromatin Isolation by RNA Purification Assay

CHIRP experiment was performed following the instruction of Dynabeads™ MyOne™ Streptavidin C1 from Invitrogen (Catalog number: 65001, Thermo Fisher Scientific, MA, USA). Briefly speaking, ovarian cancer cells (3×10^7^ cells) were collected and crosslinked with 1% glutaraldehyde solution and lysed. After treating with ultrasonic crushing, the probe purchased from RiboBio (Guangzhou, China) company was incubated with the cell lysate at 37°C for 4 h, and then magnetic beads were added to collect the probe. Then the product was collected for RNA extraction and protein extraction, separately.

### Animal Study

All animal experiments were approved by the Animal Care and Use Committee of Sichuan University (Approve number: 2019189A) following the ARRIVE guidelines 2.0. Female BALB/c nude mice (6 weeks old) were purchased from the Beijing HFK Bioscience Co., Ltd. (Beijing, China) and feed in a specific pathogen free condition of Animal Center of Sichuan University with free access to food and water. For subcutaneous tumor model establishment, control cells and MIR210HG knockout A2780 and SKOV3 cells (5×10^6^ cells) were injected into the dorsal flank of the BALB/c mice. Tumor size was determined by measuring length and width every five days with vernier caliper, and calculating the tumor volume (mm^3^) as: *V*=tumor length× (tumor width)^2^ ×0.52. When mice sacrificed, tumors were collected, weighed and used for further experiments. At the end of animal study, the mice were intraperitoneally injected with 60 μl 10% chloraldurate, and then sacrificed by breaking the neck.

### Immunofluorescence and Immunohistochemical Staining

For immunofluorescence staining in cells, A2780 and SKOV3 cells were cultured on coverslips in six-well plates and fixed with 4% paraformaldehyde for 15 minutes and permeabilized with 0.1% Triton X-100 for 15 min at room temperature (22–25°C). Primary antibodies against E-cadherin (Catalog number: 14472, CST, MA, USA, 1:200) and N-cadherin (Catalog number: 13116, CST, MA, USA, 1:200) diluted in PBS was added and incubated at 4°C for overnight. Then the slides were washed with PBS for 3 times, and incubated with Cy3-conjuated secondary antibody (Thermo Fisher, MA, USA) away from light at room temperature (22–25°C) for 1 hour. Slides were mounted with Prolong Gold antifade plus DAPI (Invitrogen) and slides were viewed with BX51 (Olympus, Tokyo, Japan).

A2780 tumor tissues were fixed in 4% Paraformaldehyde, embedded in paraffin, and then cut into 4 μm thick sections for IHC staining. After antigen retrieval in a high temperature and high pressure condition for 3 minutes, the slides were blocked with non-immune goat serum and incubated with primary antibodies against CD31 (Catalog number: 28083-1-AP, Proteintech, Wuhan, China, 1:400), HIF-1α (Catalog number: 36169, CST, MA, USA, 1:100) and VEGF (Catalog number: ab46154, Abcam, London, UK, 1:100) at 4°C for overnight. For immunofluorescence staining, after washing with PBS for 3 times, the slides were incubated with Cy3-conjuated and FITC-conjugated secondary antibody (Thermo Fisher, MA, USA) at room temperature (22–25°C) for 1 hour. Slides were mounted with Prolong Gold antifade plus DAPI (Invitrogen) and slides were viewed with BX51 (Olympus, Tokyo, Japan). The number of tumor vessels and the percent of HIF-1α positive cells in each frame was analyzed. For immunohistochemical staining, after washing with PBS for 3 times, the slides were incubated with HRP-conjugated secondary antibodies for 2 h (SP9001, Zsbio, Beijing, China) and then stained with 3,3′-diaminobenzidine (Maixin, Fuzhou, China). Cell nucleus were stained by hematoxylin (Catalog number: C0105M, Beyotime, Beijing, China).

### Clinical Samples

All human epithelial ovarian cancer tissues and paired adjacent noncancerous ovarian tissues were collected as surgical specimens at the West China Second Hospital from March, 2011 to July, 2012, and stored in liquid nitrogen. The prognosis data were collected by telephone follow-up from 2017 to 2019. The RNAs were extracted in 2020 and qPCR was performed immediately. All patients signed an informed consent and the experiments were approved by the ethics committee of Sichuan University. After the determination of MIR210HG expression by qPCR, the patients were divided into MIR210HG high group and MIR210HG low group according to the media expression of MIR210HG in all malignant samples.

### Statistical Analysis

All experiments were repeated at least three times and the data are presented as the mean ± SD. All of the data were analyzed by one-way ANOVA. Disease free survive (DFS) and Overall survive (OS) trends and curves were calculated by the Kaplan–Meier method, and differences were compared using the log-rank test. The difference was considered to be significant difference when p<0.05. All of the analysis were performed using SPSS version 13.0 (SPSS Inc, IL, USA).

## Results

### Hypoxia Induced LncRNA-MIR210HG in Ovarian Cancer

**Figure 1:**
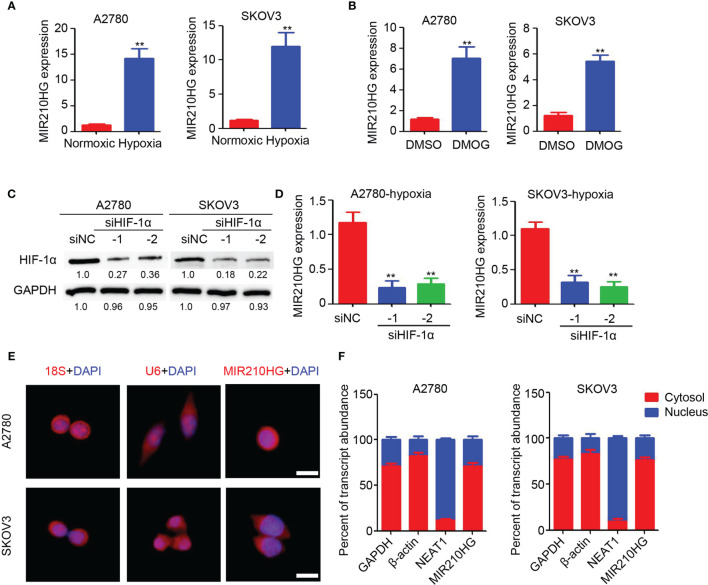
Hypoxia induced lncRNA-MIR210HG in ovarian cancer. **(A)** qPCR analysis of lncRNA-MIR210HG expression in A2780 and SKOV3 cells treated with normoxic and hypoxia condition for 48 hours (n = 3, **p < 0.01). **(B)** qPCR analysis of lncRNA-MIR210HG expression in A2780 and SKOV3 cells treated with DMSO or DMOG for 48 hours (n = 3, **p < 0.01). **(C)** Western blotting detection of HIF-1α expression in A2780 and SKOV3 cells transfected with siRNA targeting HIF-1α and negative control under hypoxia condition. GAPDH was used as a loading control. **(D)** qPCR analysis of lncRNA-MIR210HG expression in A2780 and SKOV3 cells transfected with siRNA targeting HIF-1α and negative control under hypoxia condition (n = 3, **p < 0.01). **(E)** Detection of lncRNA-MIR210HG, U6 and 18S in A2780 and SKOV3 cells by FISH staining under hypoxia condition. Cell nucleus was stained by DAPI. Scale bar = 10 μm. **(F)** qPCR analysis of lncRNA-MIR210HG, GAPDH, NEAT1 and β-actin expression in nucleus and cytosol of A2780 and SKOV3 cells under hypoxia condition.

To investigate the potential induction of hypoxia in lncRNA-MIR210HG expression, A2780 and SKOV3 cells were cultured in 1% O_2_ for 48 h. The total RNA was then collected for qPCR analysis. As shown in **Figure 1A**, significant upregulation of lncRNA-MIR210HG was observed in hypoxia-treated A2780 and SKOV3 cells, compared with cells under normoxic conditions. After treatment with DMOG for 48 h, lncRNA-MIR210HG was also dramatically increased in A2780 and SKOV3 cells (**Figure 1B**). To determine the potential role of HIF-1α in hypoxia-induced lncRNA-MIR210HG expression, siRNA targeting HIF-1α was used to transfect A2780 and SKOV3 cells, and low HIF-1α expression was observed in the cells (**Figure 1C**). qPCR analysis indicated that knockdown of HIF-1α significantly inhibited lncRNA-MIR210HG expression in A2780 and SKOV3 cells (**Figure 1D**). These results indicate that lncRNA-MIR210HG is a hypoxia-induced lncRNA in ovarian cancer, which is mediated by HIF-1α.

### Distribution of LncRNA-MIR210HG in Hypoxic Ovarian Cancer Cells

Next, the lncRNA-MIR210HG distribution was determined by FISH staining. Our results indicated that 18S was mainly located in the cell nucleus and U6 was mainly located in the cytosol (**Figure 1E**). lncRNA-MIR210HG was mainly located in the cytosol, while a less positive signal was also observed in the nucleus of ovarian cancer cells (**Figure 1E**). Furthermore, A2780 and SKOV3 cells were collected under hypoxic conditions for cell nucleus and cytosol separation. qPCR analysis indicated that NEAT1 was mainly located in the cell nucleus, which confirmed the accuracy of the operation (**Figure 1F**), and lncRNA-MIR210HG was mainly located in the cytosol, which was consistent with the FISH staining results. Collectively, lncRNA-MIR210HG was mainly located in the cytosol of hypoxic ovarian cancer cells.

### LncRNA-MIR210HG Promotes EMT in Ovarian Cancer

**Figure 2:**
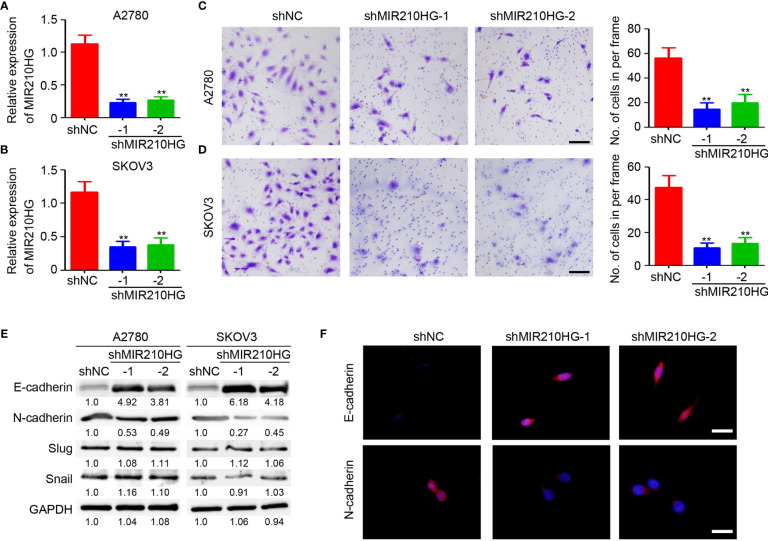
lncRNA-MIR210HG promotes EMT in ovarian cancer. **(A, B)** qPCR analysis of lncRNA-MIR210HG expression in A2780-shNC, A2780-shMIR210HG, SKOV3-shNC and SKOV3-shMIR210HG cells under hypoxia condition (n = 3, **p < 0.01). **(C, D)** A2780-shNC, A2780-shMIR210HG, SKOV3-shNC and SKOV3-shMIR210HG cells were used for Matrigel-based migration assay under hypoxia condition. The number of migrated cells in per frame was analyzed (n = 4, **p < 0.01, Scale bar = 100 μm). **(E)** Detection of E-cadherin and N-cadherin expression in A2780-shNC, A2780-shMIR210HG, SKOV3-shNC and SKOV3-shMIR210HG cells that were cultured under hypoxia condition by western blotting. GAPDH was used as a loading control. **(F)** Detection of E-cadherin and N-cadherin expression in A2780-shNC and A2780-shMIR210HG cells that were cultured under hypoxia condition by immunofluorescent staining (Scale bar = 10 μm).

Epithelial-mesenchymal transition (EMT) is an important process under hypoxic conditions. Thus, we investigated the potential function of lncRNA-MIR210HG in regulating EMT in ovarian cancer. Lentivirus-based shRNA targeting lncRNA-MIR210HG was employed to infect A2780 and SKOV3 cells and the stably infected cells were collected for further analysis. qPCR analysis confirmed the efficient knockdown or lncRNA-MIR210HG in A2780-shMIR210HG and SKOV3-shMIR210HG cells (**Figures 2A, B**). The cells were then used for a Matrigel-based migration assay under hypoxic conditions. Our results indicated that knockdown of lncRNA-MIR210HG significantly inhibited the migration of A2780 (**Figure 2C**) and SKOV3 (**Figure 2D**) cells. Western blotting results demonstrated that knockdown or lncRNA-MIR210HG promoted E-cadherin expression and inhibited N-cadherin expression both in A2780 and SKOV3 cells (**Figure 2E**), which was also confirmed by immunofluorescence staining (**Figure 2F**). Collectively, knockdown of lncRNA-MIR210HG inhibits EMT in ovarian cancers under hypoxic conditions.

### LncRNA-MIR210HG Promotes Angiogenesis in Ovarian Cancer

**Figure 3:**
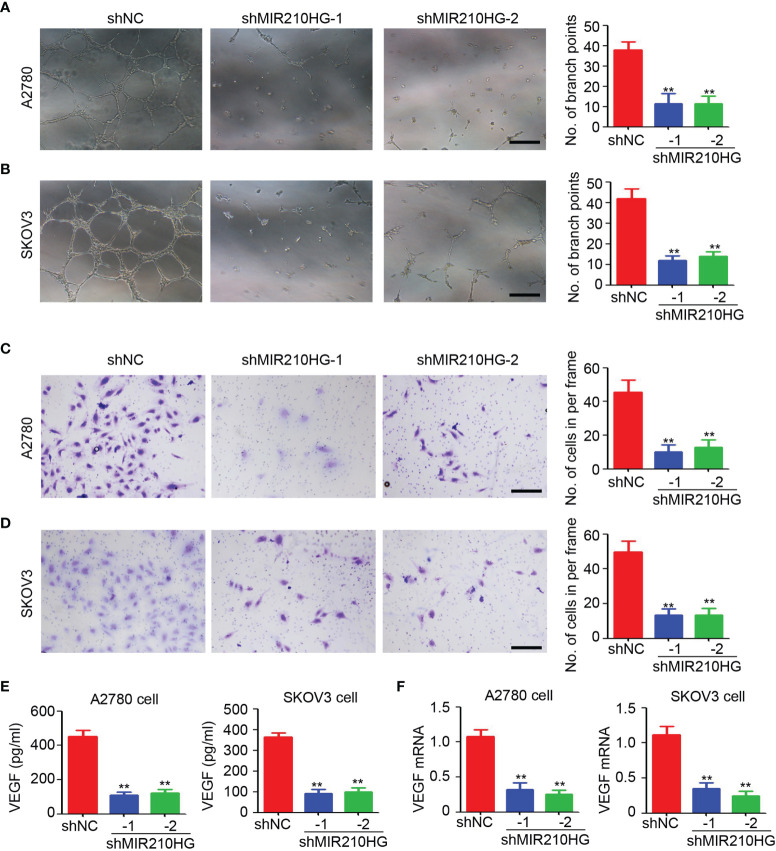
lncRNA-MIR210HG promotes angiogenesis in ovarian cancer. **(A, B)** The conditional culture medium from A2780-shNC, A2780-shMIR210HG, SKOV3-shNC and SKOV3-shMIR210HG cells that were cultured under hypoxia condition were collected for HUVECs tube formation assay. The number of branch points formed in each frame was counted and analyzed (n = 4, **p < 0.01, Scale bar = 100 μm). **(C, D)** The conditional culture medium from A2780-shNC, A2780-shMIR210HG, SKOV3-shNC and SKOV3-shMIR210HG cells that were cultured under hypoxia condition were collected for HUVECs migration assay. The number of migrated cells in each frame was counted and analyzed (n = 4, **p < 0.01, Scale bar = 100 μm). **(E)** ELISA detection of VEGF expression in conditional culture medium from A2780-shNC, A2780-shMIR210HG, SKOV3-shNC and SKOV3-shMIR210HG cells that were cultured under hypoxia condition (n = 3, **p < 0.01). **(F)** qPCR analysis of VEGF mRNA expression in A2780-shNC, A2780-shMIR210HG, SKOV3-shNC and SKOV3-shMIR210HG cells that were cultured under hypoxia condition (n = 3, **p < 0.01).

To determine the potential function of lncRNA-MIR210HG in tumor angiogenesis, the conditional culture medium from A2780 and SKOV3 cells under hypoxic conditions were collected for HUVEC (human umbilical vein endothelial cell)-based tube formation and migration assays. As shown in **Figures 3A, B**, fewer tubes were observed in HUVECs treated with conditional culture medium from A2780-shMIR210HG and SKOV3-shMIR210HG cells. Analysis of branch points per frame confirmed the significant inhibition of tube formation in the lncRNA-MIR210HG knockdown group (**Figures 3A, B**). Further results also suggested that the conditional culture medium from the lncRNA-MIR210HG knockdown group dramatically inhibited the migration of HUVECs (**Figures 3C, D**). ELISA was employed to determine VEGF expression in conditional culture medium and demonstrated that knockdown of lncRNA-MIR210HG significantly inhibited VEGF secretion in both A2780 and SKOV3 cells (**Figure 3E**). A similar reduction in VEGF mRNA was also observed in A2780-shMIR210HG and SKOV3-shMIR210HG cells (**Figure 3F**). These results indicated that knockdown of lncRNA-MIR210HG inhibits tumor angiogenesis in ovarian cancer.

### LncRNA-MIR210HG Inhibits HIF-1α Degradation in Ovarian Cancer

**Figure 4:**
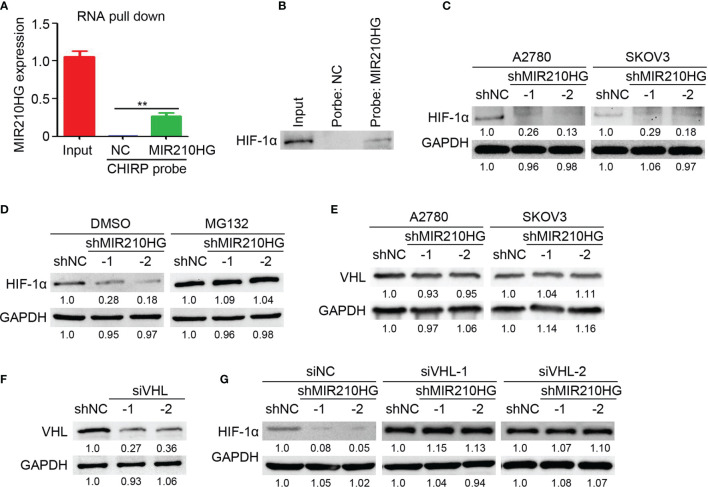
lncRNA-MIR210HG inhibits HIF-1α degradation in ovarian cancer. **(A)** A2780 cells that were cultured under hypoxia condition were collected for RNA pull down experiments with CHIRP probe targeting lncRNA-MIR210HG and negative control. qPCR analysis of lncRNA-MIR210HG expression in A2780 cells (Input) and products of RNA pull down (n = 3, **p < 0.01). **(B)** Detection of HIF-1α expression in A2780 cells (Input) and products of RNA pull down. **(C)** Detection of HIF-1α expression in A2780 and SKOV3 cells (under hypoxia condition) stably infected with lentivirus-shRNA targeting lncRNA-MIR210HG and negative control by western blotting. GAPDH was used as a loading control. **(D)** Detection of HIF-1α expression in A2780-shNC and A2780-shMIR210HG cells (under hypoxia condition) treated with DMSO or MG132 by western blotting. GAPDH was used as a loading control. **(E)** Detection of VHL expression in A2780-shNC, A2780-shMIR210HG, SKOV3-shNC and SKOV3-shMIR210HG cells (under hypoxia condition) by western blotting. GAPDH was used as a loading control. **(F)** Detection of VHL expression in A2780 cells (under hypoxia condition) transfected with siRNA targeting VHL and negative control by western blotting. GAPDH was used as a loading control. **(G)** Detection of HIF-1α expression in A2780-shNC and A2780-shMIR210HG cells (under hypoxia condition) transfected with siRNA targeting VHL and negative control. GAPDH was used as a loading control.

To determine the direct target of lncRNA-MIR210HG in ovarian cancer, RNA pull-down was performed with a CHIRP probe targeting lncRNA-MIR210HG. Our results indicated that lncRNA-MIR210HG could be efficiently pulled down by the CHIRP probe (**Figure 4A**). We tried to detect HIF-1α expression in the products of RNA pull-down experiments by western blotting and was surprised to detect HIF-1α in the production of RNA pull-down of the CHIRP-MIR210HG probe (**Figure 4B**). Further results indicated that knockdown of lncRNA-MIR210HG inhibited HIF-1α expression in both A2780 and SKOV3 cells (**Figure 4C**). Blocking of protease activity by MG132 efficiently attenuated the reduction of HIF-1α expression in A2780-shMIR210HG cells, suggesting that lncRNA-MIR210HG regulated HIF-1α expression was protease dependent (**Figure 4D**). VHL was the crucial protease in promoting HIF-1α degradation. Thus, we determined the expression of VHL in A2780 and SKOV3 cells and found that lncRNA-MIR210HG had little effect on VHL expression in ovarian cancer cells (**Figure 4E**). Furthermore, knockdown of VHL by siVHL transfection also efficiently blocked shMIR210HG-mediated HIF-1α reduction in ovarian cancer cells (**Figures 4F, G**). These results demonstrated that lncRNA-MIR210HG directly targets and regulates HIF-1α expression in a VHL-dependent manner.

### Knockdown of LncRNA-MIR210HG Inhibits Tumor Growth in Mice

**Figure 5:**
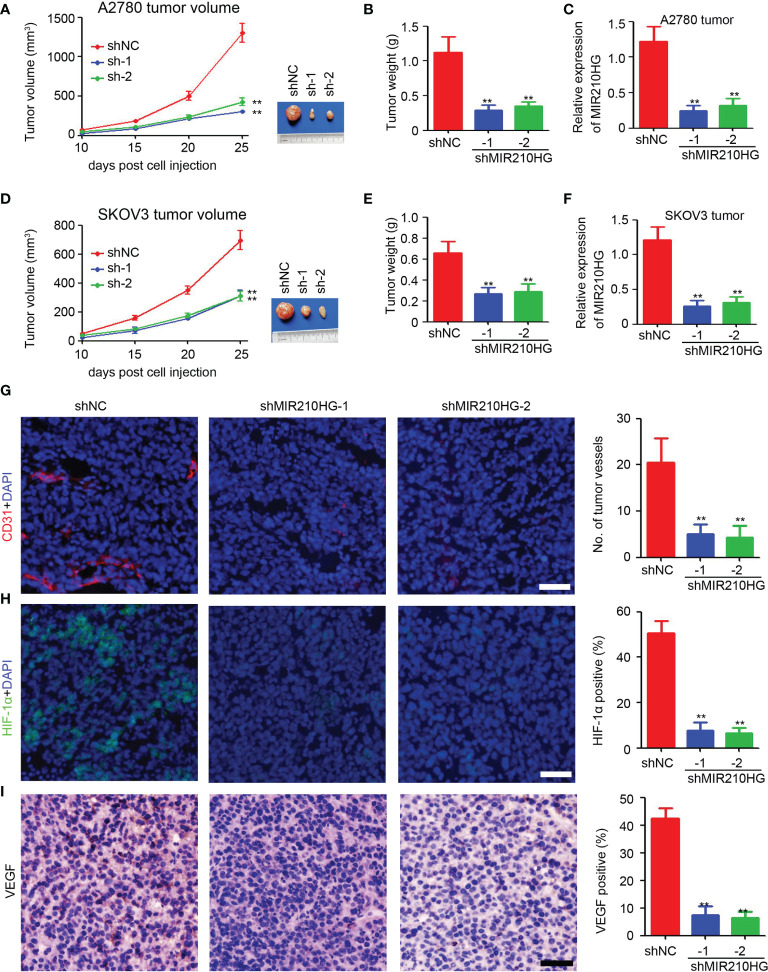
Knockdown of lncRNA-MIR210HG inhibits tumor growth in mice. **(A)** A2780-shNC and A2780-shMIR210HG cells were injected into nude mice to establish subcutaneous tumor model. Tumor volume was measured every 5 days and the tumor growth curve was performed (n = 5, **p < 0.01). **(B)** Tumor weight of A2780-shNC and A2780-shMIR210HG tumors (n = 5, **p < 0.01). **(C)** qPCR analysis of lncRNA-MIR210HG expression in A2780-shNC and A2780-shMIR210HG tumors (n = 3, **p < 0.01). **(D)** SKOV3-shNC and SKOV3-shMIR210HG cells were injected into nude mice to establish subcutaneous tumor model. Tumor volume was measured every 5 days and the tumor growth curve was performed (n = 5, **p < 0.01). **(E)** Tumor weight of SKOV3-shNC and SKOV3-shMIR210HG tumors (n = 5, **p < 0.01). **(F)** qPCR analysis of lncRNA-MIR210HG expression in SKOV3-shNC and SKOV3-shMIR210HG tumors (n = 3, **p < 0.01). **(G)** Detection of tumor angiogenesis in A2780-shNC and A2780-shMIR210HG tumors by CD31 staining. Cell nucleus was stained with DAPI. The number of tumor vessels was analyzed (n = 3, **p < 0.01, Scale bar = 100 μm). **(H)** Detection of HIF-1α in A2780-shNC and A2780-shMIR210HG tumors by immunofluorescent staining. Cell nucleus was stained with DAPI. The percent of HIF-1α positive cells was analyzed (n = 3, **p < 0.01, Scale bar = 100 μm). **(I)** Detection of VEGF in A2780-shNC and A2780-shMIR210HG tumors by IHC staining. The percent of VEGF positive cells was analyzed (n = 3, **p < 0.01, Scale bar = 100 μm).

To determine the potential role of lncRNA-MIR210HG in tumor growth in mice, ovarian cancer cells were injected to establish a subcutaneous tumor model. As shown in **Figures 5A, B**, knockdown of lncRNA-MIR210HG significantly inhibited A2780 tumor growth with a 76.7% and 67.1% reduction in tumor volume (A2780-shNC group: 1302.5 ± 261.9 mm^3^ vs. shMIR210HG-1 group: 303.6 ± 46.5 mm^3^ vs. shMIR210HG-2 group: 428.6 ± 102.0 mm^3^). A similar reduction in tumor weight was also observed in A2780-shMIR210HG-1 (73.9% reduction) and A2780-shMIR210HG-2 (68.9% reduction) tumors (**Figure 5B**). qPCR analysis confirmed the significant downregulation of lncRNA-MIR210HG in A2780-shMIR210HG-1 and A2780-shMIR210HG-2 tumors (**Figure 5C**). We also observed a significant inhibition of tumor growth in SKOV3-shMIR210HG-1 (55.0% reduction in tumor volume and 59.7% reduction in tumor weight) and SKOV3-shMIR210HG-2 (55.4% reduction in tumor volume and 56.1% reduction in tumor weight) tumors (**Figures 5D, E**). qPCR analysis confirmed the significant downregulation of lncRNA-MIR210HG in SKOV3-shMIR210HG-1 and SKOV3-shMIR210HG-2 tumors (**Figure 5F**). Further staining indicated that knockdown of lncRNA-MIR210HG significantly inhibited tumor angiogenesis in A2780 tumors (**Figure 5G**). Less HIF-1α positive and VEGF positive cells were also detected in A2780-shMIR210HG-1 and A2780-shMIR210HG-2 tumors (**Figures 5H, I**). Collectively, knockdown of lncRNA-MIR210HG impairs tumor growth in mice by inhibiting tumor angiogenesis and HIF-1α/VEGF expression.

### Upregulation of LncRNA-MIR210HG Predicts Poor Prognosis of Ovarian Cancer Patients

**Figure 6:**
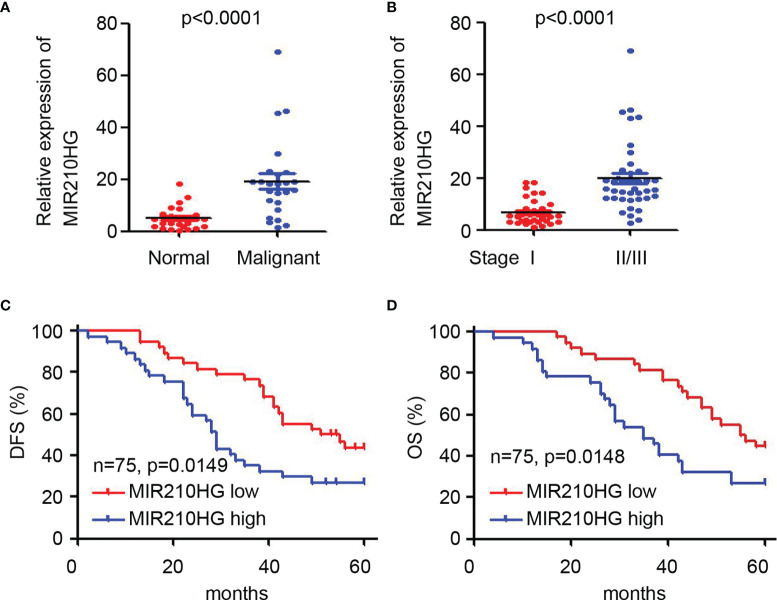
Upregulation of lncRNA-MIR210HG predicts poor prognosis of ovarian cancer patients. **(A)** qPCR analysis of lncRNA-MIR210HG expression in adjacent normal and malignant tissues of ovarian cancer patients. **(B)** qPCR analysis of lncRNA-MIR210HG expression in malignant tissues from stage I or stage II/III ovarian cancer patients. **(C, D)** Ovarian cancer patients were dived into lncRNA-MIR210HG high and low expression group based on the median expression of lncRNA-MIR210HG expression in 75 malignant tissues. Analysis of disease-free survival (DFS) and overall survival (OS) time in the lncRNA-MIR210HG high and low group.

To determine the potential clinical correlation of lncRNA-MIR210HG in ovarian cancer, qPCR was employed to detect lncRNA-MIR210HG expression in 75 malignant tissues (**Table 1**) and 25 adjacent normal tissues. Our results suggested that lncRNA-MIR210HG was significantly upregulated in ovarian cancer tissues compared with adjacent normal tissues (**Figure 6A**). Further analysis indicated that malignant tissues from stage II/III ovarian cancer patients had higher lncRNA-MIR210HG expression than stage I ovarian cancer patients (**Figure 6B**). Next, the 75 ovarian cancer patients were divided into lncRNA-MIR210HG high and low expression groups based on the median lncRNA-MIR210HG expression in malignant tissues. Patients with low lncRNA-MIR210HG expression had a longer disease-free survival (**Figure 6C**) and overall survival (**Figure 6D**). These results suggest that upregulation of lncRNA-MIR210HG in ovarian cancer tissues is correlated with cancer progression and poor prognosis of patients.

**Table 1:** Baseline characteristics of patients (N = 75).

Characteristic	Number of cases (N = 75)	Constituent ratio (%)
Age-yr		
Median	64	-
Range	56-76	-
Sex		
Male	0	0
Female	75	100
TNM stage		
I	33	44
II/III	42	56

## Discussion

Here, we demonstrated that MIR210HG was induced by hypoxia, which is HIF-1α dependent and is mainly located in the cytosol of ovarian cancer cells. Knockdown of MIR210HG significantly inhibited EMT and tumor angiogenesis *in vitro* and impaired tumor growth in mice. Molecular investigations indicated that MIR210HG directly targets HIF-1α and inhibits VHL-dependent HIF-1α degradation in ovarian cancer. Further results demonstrated that MIR210HG was upregulated in ovarian cancer tissues and correlated with tumor progression and poor prognosis of ovarian cancer patients. Our study suggests that hypoxia-induced MIR210HG promotes cancer progression by inhibiting HIF-1α degradation in ovarian cancer, which could be a therapeutic target for ovarian cancer.

MIR210HG is a de-expressed lncRNA in diverse cancers. In hepatocellular carcinoma, MIR210HG expression was increased in malignant tissues and cancer cells compared with that in paired adjacent normal liver tissue samples and normal liver cell lines, respectively (26). High MIR210HG expression was correlated with advanced clinical stage, large tumor size, present vascular invasion, and unfavorable histological differentiation of hepatocellular carcinoma (26). MIR210HG is highly expressed in NSCLC tissues and its expression is correlated with tumor stage and lymph node metastasis in NSCLC patients (21). Furthermore, MIR210HG expression level was significantly upregulated in osteosarcoma tissue samples, invasive breast cancer cells, colorectal cancer cells, and the aberrantly enhanced MIR210HG expression predicted poor prognosis and low survival rate of cancer patients (19, 22–25, 28). Here, we first demonstrated that the upregulation of MIR210HG in malignant tissues of ovarian cancer patients and MIR210HG high expression was correlated with tumor progression and poor prognosis of ovarian cancer patients, which suggested that MIR210HG is a potential prognostic predictor for ovarian cancer patients. Our results also indicated that MIR210HG was a hypoxia-induced lncRNA, which is consistent with previous studies (29). However, we demonstrated that MIR210HG induction under hypoxic conditions is HIF-1α dependent. Further investigations are needed to clarify the underlying mechanism by which HIF-1α induces MIR210HG expression in ovarian cancer.

The amount of lncRNAs contributed to the progression of ovarian cancer. LncRNA-pro-transition associated RNA (PTAR) promotes EMT, invasion-metastasis, and tumorigenicity in serous ovarian cancer (30). LncRNA-plasmacytoma variant translocation I (PVT1), metastasis-associated lung adenocarcinoma transcript 1 (MALAT1), and LINC00319 were demonstrated to be involved in ovarian cancer cell proliferation, migration, and invasion by acting as a ceRNA (31–33). MIR210HG was also an important regulator of cell proliferation and invasion in diverse cancers (19, 20, 22, 23). Our study first determined the function of MIR210HG in ovarian cancer and confirmed the promoting role of MIR210HG in EMT and tumor angiogenesis in ovarian cancer. *In vivo* results also provided solid evidence for clarifying the tumor growth inhibition in MIR210HG knockdown ovarian cancer cells, accompanied by inhibition of tumor angiogenesis. All of the results first clarified the function of MIR210HG in ovarian cancer and provided a therapeutic target for ovarian and other cancers.

Previous studies indicated that MIR210HG is a competing endogenous RNA (ceRNA) of miR-503-5p, miR-874, miR-503, miR-1226-3p in cervical cancer (18), NSCLC (20), osteosarcoma cell (22), and breast cancer (19). MIR210HG could recruit DNMT1, thereby promoting methylation of the CACNA2D2 promoter region in NSCLC (21). In triple-negative breast cancer, MIR210HG potentiated the metabolic transcription factor HIF-1α translation *via* directly binding to the 5’-UTR of HIF-1α mRNA, leading to increased HIF-1a protein level (34). Here, RNA pull-down results demonstrated that MIR210HG directly targets HIF-1α protein in ovarian cancer cells under hypoxic conditions. Knockdown of MIR210HG inhibited HIF-1α protein expression in a protease dependent manner. Knockdown of VHL also efficiently blocked shMIR210HG-mediated HIF-1α reduction in ovarian cancer cells, which suggested that MIR210HG regulates HIF-1α expression in a VHL-dependent manner. These results clarified the binding protein of MIR210HG, but further investigations are needed to clarify the binding site between MIR210HG and HIF-1α protein.

## Conclusion

Collectively, the present study provides solid evidence for understanding the expression and function of MIR210HG in ovarian cancer, which may be a potential therapeutic target for ovarian cancer. However, further investigations are needed to clarify the underlying mechanism by which HIF-1α induces MIR210HG expression in ovarian cancer.

## Data Availability Statement

The original contributions presented in the study are included in the article/supplementary material. Further inquiries can be directed to the corresponding author.

## Ethics Statement

The studies involving human participants were reviewed and approved by the ethics committee of Sichuan University. The patients/participants provided their written informed consent to participate in this study. The animal study was reviewed and approved by the Animal Care and Use Committee of Sichuan University.

## Author Contributions

PL, HH, XQ, CB, and MC conducted all the experiments. YQ participated in the design of the study and helped to draft the manuscript. LL and LX conducted the statistical analysis. XZ and TY designed the project and finalized the manuscript. All authors read and approved the final manuscript.

## Funding

This study was supported by the National Science and Technology Major Projects for Major New Drugs Innovation and Development of China (2018ZX09733-001-004) and the Fundamental Research Funds for the Central Universities (SCU2020D4132).

## Conflict of Interest

The authors declare that the research was conducted in the absence of any commercial or financial relationships that could be construed as a potential conflict of interest.

## Publisher’s Note

All claims expressed in this article are solely those of the authors and do not necessarily represent those of their affiliated organizations, or those of the publisher, the editors and the reviewers. Any product that may be evaluated in this article, or claim that may be made by its manufacturer, is not guaranteed or endorsed by the publisher.
